# The weaponization of medicine: Early psychosis in the Black community and the need for racially informed mental healthcare

**DOI:** 10.3389/fpsyt.2023.1098292

**Published:** 2023-02-09

**Authors:** Sonya C. Faber, Anjalika Khanna Roy, Timothy I. Michaels, Monnica T. Williams

**Affiliations:** ^1^Department of Epidemiology and Public Health, University of Ottawa, Ottawa, ON, Canada; ^2^Counselling Psychology, Faculty of Education, University of Ottawa, Ottawa, ON, Canada; ^3^Department of Psychiatry Research, Zucker Hillside Hospital, Northwell Health, Glen Oaks, NY, United States; ^4^Donald and Barbara Zucker School of Medicine at Hofstra/Northwell Health, Hempstead, NY, United States; ^5^Department of Psychology, University of Ottawa, Ottawa, ON, Canada

**Keywords:** psychosis, Black communities, discrimination, racism, misdiagnosis, weaponization of medicine, schizophrenia

## Abstract

There is a notable disparity between the observed prevalence of schizophrenia-spectrum disorders in racialized persons in the United States and Canada and White individuals in these same countries, with Black people being diagnosed at higher rates than other groups. The consequences thereof bring a progression of lifelong punitive societal implications, including reduced opportunities, substandard care, increased contact with the legal system, and criminalization. Other psychological conditions do not show such a wide racial gap as a schizophrenia-spectrum disorder diagnosis. New data show that the differences are not likely to be genetic, but rather societal in origin. Using real-life examples, we discuss how overdiagnoses are largely rooted in the racial biases of clinicians and compounded by higher rates of traumatizing stressors among Black people due to racism. The forgotten history of psychosis in psychology is highlighted to help explain disparities in light of the relevant historical context. We demonstrate how misunderstanding race confounds attempts to diagnose and treat schizophrenia-spectrum disorders in Black individuals. A lack of culturally informed clinicians exacerbates problems, and implicit biases prevent Black patients from receiving proper treatment from mainly White mental healthcare professionals, which can be observed as a lack of empathy. Finally, we consider the role of law enforcement as stereotypes combined with psychotic symptoms may put these patients in danger of police violence and premature mortality. Improving treatment outcomes requires an understanding of the role of psychology in perpetuating racism in healthcare and pathological stereotypes. Increased awareness and training can improve the plight of Black people with severe mental health disorders. Essential steps necessary at multiple levels to address these issues are discussed.

## 1. Introduction

In the words of Martin Luther King Jr., “*Of all the forms of inequality, injustice in health is the most shocking and the most inhuman…”* (1).

## 2. Psychotic disorders in the United States and Canada

*Psychosis* is understood broadly to refer to mental states that involve a disconnect from reality. Positive symptoms include hallucinations (perceiving things that are not present, which can be auditory, tactile, visual, olfactory, or taste), delusions (false beliefs that are not easily changed), and disorganized thinking, speech, or behavior (2). Negative symptoms include difficulties with emotional and facial expressions, speech, thinking, task commencement, motivation, and social withdrawal. Other symptoms include cognitive deficits. Psychosis can be a symptom of many different mental and physical disorders such as schizophrenia as well as bipolar disorder and major depressive disorder (MDD), so there is no one simple course, presentation, or cause. Rather, research indicates that a psychotic episode can be brought on by a combination of factors. Biological factors, including genetic factors from genome-wide studies, emphasize the connections between common mental disorders and genes that regulate specific neuronal functions (3, 4). While genetic factors may increase the risk of experiencing psychosis, it is environmental stressors [such as traumatic life events, autoimmune insult, or substance use; (3, 4)] that often act as the trigger and barriers to care such as lack of knowledge about the symptoms, lack of possibilities for treatment (i.e., no local specialists), and lack of access (i.e., no insurance) which can exacerbate the condition.

Therefore, it is imperative that disorders that include psychosis are correctly diagnosed with adequate consideration of environmental factors, to receive appropriate care and early intervention, which can improve prognosis. In this paper, the term “psychotic disorders” will refer broadly to all schizophrenia-spectrum disorders and other conditions that may include psychotic episodes (e.g., bipolar disorder and severe MDD), whereas the term “schizophrenia-spectrum disorders” refers to all disorders in this category, as per the DSM-5. In the interest of space, we will not be focusing on MDD or bipolar disorder, although these disorders may include psychosis, and as such, many of the same issues still apply.

The literature suggests that schizophrenia-spectrum disorders affect up to 4% of the population (5), and for the subset of those affected by schizophrenia, the global prevalence is 1 in 300 people (0.32%) or 24 million people, as shown in Table 1 (6). In the United States, schizophrenia similarly affects 0.25–0.65% of the population [~2.6 million adults aged 18 years and older; (6, 7)], and 40% of individuals with schizophrenia go untreated in any given year. However, counting those with schizophrenia in the United States is complicated, and there is an undercount because so many are incarcerated, homeless, suffer an early death, or are otherwise excluded from official treatment channels. Therefore, a more accurate count based on the 2020 census reaches 1.62%, or 3.8 million adults, which is significantly higher than previous prevalence estimates (7).

**Table 1:** Global prevalence and age-standardized prevalence for schizophrenia in 1990 and 2019 (6).

**1990**		**2019**	
**Prevalence, in millions (95% UI)**	**Age-standardized prevalence per 100,000 people (95% UI)**	**Prevalence, in millions (95% UI)**	**Age-standardized prevalence per 100,000 people (95% UI)**
Total	14_·_2 (12_·_2–16_·_5)	289_·_9 (249_·_8–333_·_2)	23_·_6 (20_·_2–27_·_2)
Male	7_·_5 (6_·_4–8_·_7)	304_·_5 (262_·_6–350_·_0)	12_·_4 (10_·_6–14_·_3)
Female	6_·_7 (5_·_8–7_·_7)	274_·_9 (236_·_9–315_·_5)	11_·_2 (9_·_6–12_·_9)

The development of schizophrenia-spectrum disorders has been found to vary by gender, with men experiencing the onset of symptoms earlier (late teens to early twenties) and women experiencing them later (early twenties to early thirties). Individuals with a family history of mental illness are at a higher risk of developing schizophrenia-spectrum disorders (8). The early adulthood or late adolescence onset of schizophrenia is particularly tragic as it negatively affects the trajectory of young people as they are poised to embark on an independent life. Globally, schizophrenia is among the top ten causes of disability-adjusted life-years. The annual costs in Canada of schizophrenia are estimated to reach up to Canadian $10 billion (5), while in the United States, the staggering direct and indirect costs of schizophrenia reached $281.6 billion in 2020. A large proportion of the direct costs are derived from healthcare, homelessness, incarceration, and necessary housing of these patients. The total lifetime economic cost of each individual diagnosed with schizophrenia at the age of twenty-five years comes to approximately $92,000 annually (9). These numbers testify to the urgent need for better care in this area.

## 3. Race and psychology (psychosis in the Black community)

In the United States and Canada, Black persons include those who are racialized as African American, Black American, Caribbean Black, and Black African, and may originate from any country. Persons racialized as Black typically have darker skin shades but may have any color of skin. Black is a racial grouping defined by the federal government (US Census Bureau, Statistics Canada) and is not the same as an ethnic group, nor synonymous with biological relatedness. For this study, Black refers to people racialized as Black by American and Canadian society who have some African ancestry.

**Figure 1:**
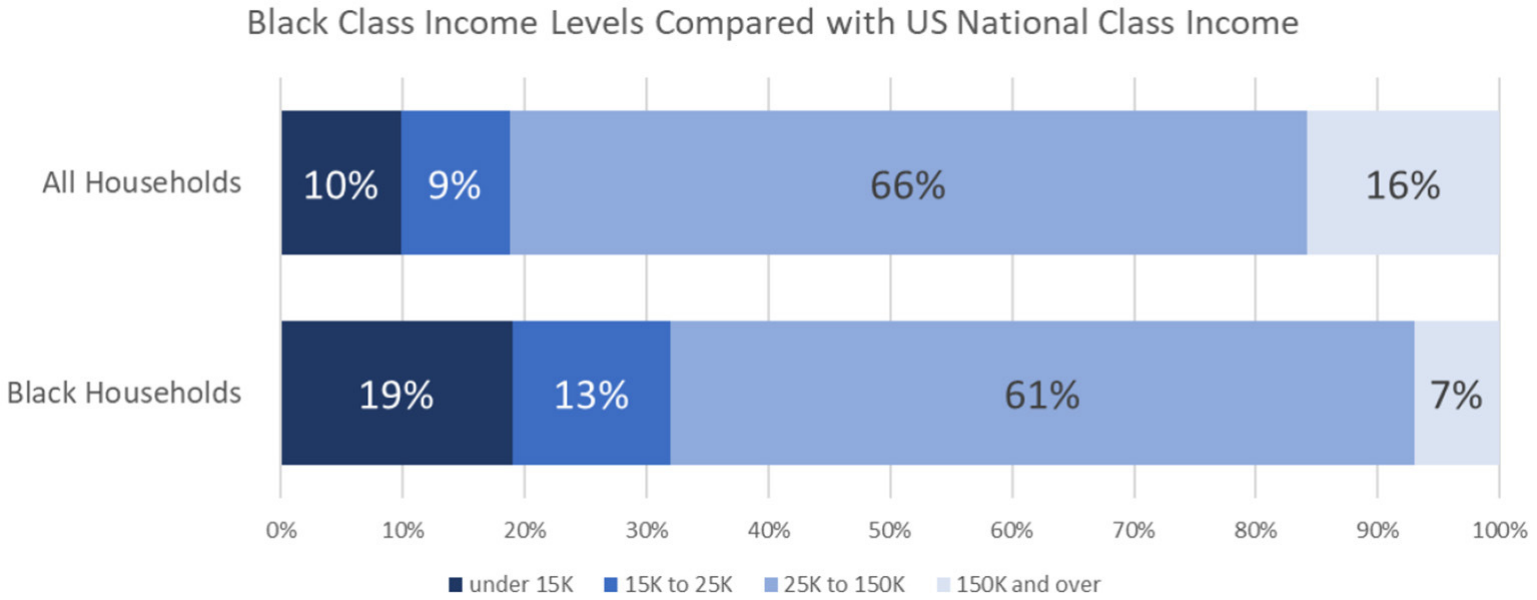
Socioeconomic class of Black households in the United States as compared with national class divisions.

In any conversation about racial disparities, it is common to wonder if observed differences are a reflection of socioeconomic differences (class and income). Although people of different classes may be treated differently in the healthcare system, the issue we are highlighting in this paper is specifically about Black people. Contrary to popular stereotypes, most Black people in the United States and Canada are middle class, not poor [81% of Black Americans are not in poverty; Figure 1; (10)]. Certainly, Black people are overrepresented among the poor, but this is due to racism, which takes us back to the issue of race, rather than socioeconomic status.

Similarly, there are pathological stereotypes of Black people surrounding increased substance use, dysfunctional parenting, and poor social skills—which some might assume are factors in the development and maintenance of schizophrenia-spectrum disorders in this population. Addressing these false beliefs are beyond the scope of this paper; however, interested readers are directed to Williams et al. (11) and Jahn et al. (12) studies.

### 3.1. Epidemiology of race and psychosis

A national epidemiological study by Cohen and Marino (13) found that Black Americans had higher lifetime rates of disorders that included psychotic symptoms (15.3%) compared with Latino (13.6%), White (9.7%), and Asian Americans (9.6%). Furthermore, the lifetime probability of these disorders was associated with the Black and Hispanic race, the current level of disability, and a lifetime diagnosis of substance use disorder or posttraumatic stress disorder (PTSD). African American/Black communities are diagnosed with schizophrenia-spectrum disorders at a rate that is three to four times higher than White communities (14). Studies have shown that Black Americans are 2.4 times more likely to be diagnosed with schizophrenia (13) and are more likely to be diagnosed with schizophrenia-paranoid subtype or schizophrenia-undifferentiated subtype (14, 15). As will be further discussed, some of these disparities are due to clinician error, including overdiagnosis and misdiagnosis.

Finally, for Black people experiencing early symptoms of psychotic disorders, contact with law enforcement often represents the entry point into the medical system and is occurring even in the premorbid and prodromal stages (16). This means that these individuals have a high risk of becoming a target of law enforcement, due to negative stereotypes about Black people, which brings a host of negative life consequences (11, 17). Having been diagnosed with both a serious mental illness and a history of incarceration makes gainful employment nearly impossible. Furthermore, under United States laws, having a criminal record excludes both the patient and their family (if they live together) from assistance for public housing. Taken together, these inhumane and punitive barriers are a recipe for homelessness and marginalization and virtually ensure that first-episode patients will be unable to establish independent lives (16, 17).

### 3.2. Racism and psychosis

Black individuals suffering from psychotic disorders may be unable to access treatment or outright punished for becoming ill (Table 2). A case example, with which the authors were involved, is that of Grace Terry (pseudonym), a Black woman with schizophrenia who was incarcerated in the United States for allegedly evading arrest when frightened by police officers. In another example in Canada, Samuel Uko's family described that his race impacted how he was treated during his mental health crisis, leading to a fatal outcome. The CEO of the Saskatchewan Health Authority offered a formal apology, admitting that the staff spent too much time focusing on his identity rather than his care (18).

**Table 2:** Case study: The focus on identity rather than care.

**Description**	**Common issues arising in care pathways for psychosis**
In 2018, a 40-year-old Black Liberian American immigrant woman, Ms. Terry, was crossing a parking lot at night in a location where police were said to harass members of her community. When they approached her, she became frightened and ran, at one point, hiding behind a bystander and trying to ward off officers with an umbrella. The umbrella made contact with one of the officers, and although police photos showed no visible evidence that the White officer was harmed, Ms. Terry faced a slew of charges, including trespassing, evasion, and assault. Her initial evaluation by a forensic psychologist missed her serious mental illness—despite a medical history of psychosis and hospitalization–and the resulting report was not culturally informed. Once Ms. Terry was able to obtain a culturally informed assessment, she was found to have moderate schizophrenia—which included symptoms of paranoia, auditory hallucinations, and delusional beliefs—as well as mild intellectual disability. She ended up being incarcerated for nearly a year before the court agreed she could be released and offered the proper mental healthcare and social support.	*Law enforcement is uninformed as to how to address acute mental health crises*. *Lack of culturally informed services resulted in misdiagnosis*. *Lack of empathy and misconception of threat-level led to unnecessary, non-therapeutic incarceration*.
In 2020, security officers removed Mr. Uko, a young Black Canadian from the emergency room, who came to the Regina General Hospital twice on the same day begging for acute help for symptoms of psychosis. Mr. Uko's family said that he had been acting in a paranoid manner and had been hearing voices. He did not receive the essential patient–clinic engagement that he needed, despite the fact that he disclosed thoughts of committing suicide and that he had had one previous attempt. When he arrived at the hospital for the second time, a security officer misunderstood instructions that Mr. Uko needed to be moved, as instructions that he needed to be removed. He was forcibly removed from the hospital by four security officers without any registration or seeing a triage nurse and was found dead in Wascana Lake an hour later (18).	*Law enforcement used to address acute mental health crises*. *Racial bias de-prioritizes the needs of Black patients*. *Law enforcement uninformed as to how to address the acute mental health crisis*.

The situation today is that many Black people die because they do not have access to mental health services, but also because even when they have access they experience additional barriers once they are in the mental health services system (16). As a result of tragedies like this, there has been a public outcry from Black people across North America, noting a scarcity of providers representative of their communities and a lack of cultural competence in care [e.g., (19–21)].

### 3.3. Purpose of this paper

Racial disparities persist in mental health due to issues that include access to treatment and quality of care. Despite calls for cultural sensitivity and updates of diagnostic manuals, significant issues persist in diagnosis and treatment, especially in the case of psychotic disorders (14, 16, 22). Black individuals may have increased vulnerability to these disorders due to experiencing unique environmental stressors, such as alienation, discrimination, and racism, which contribute to etiology. Due to the double stigma of racism and having a serious mental illness, they are more likely to be marginalized and victimized by law enforcement.

The purpose of this study is to provide an overview of the experiences of Black people living in the United States and Canada with early schizophrenia-spectrum disorders and to discuss racially informed solutions for equitable mental healthcare. This study first describes racial differences in the diagnosis of schizophrenia-spectrum disorders; second, explains barriers to the treatment for Black communities; third, explains the role of bias and clinician empathy in misdiagnosis; fourth, describes the dangers of law enforcement involvement in mental health emergencies; and finally discusses solutions to address these disparities.

## 4. Medical racism and psychosis

### 4.1. Defining racism

To understand the connections among race, racism, racial discrimination, and psychosis, it is necessary to understand the terminology being used. *Race* is not a biological construct, but rather a sociopolitical construct that has no relevant genetic basis. The term *racism* refers to a system of beliefs (racial prejudices), practices (racial discrimination), and policies based on race that advantages individuals with historic power in most of the Western nations, including White people in the United States and Canada. In these societies, race categorizes people based on similar physical and social features, operating as a social caste system (23–26). *Racial discrimination* may be overt or covert. It ranges from microaggressions (everyday slights, either intentional or unintentional, that are based on race) to deprivation of societal goods or even acts of violence. Racial discrimination can be perpetrated by anyone, regardless of race or conscious intention (24, 26).

### 4.2. Implicit biases and empathy

It is important to understand that race, culture, ethnicity, and religion influence people's mental health and the need to be a part of an honest and healthy dialogue between a healthcare professional and their client or patient (26). However, conversations about race, racism, and racial disparities are difficult for many [e.g., (27, 28)]. In the case of psychotic disorders, the experience of a Black client can, for reasons explored in this study, differ drastically from those of White clients. If asked, Black clients may have harrowing tales about their experiences being stigmatized, harassed, or surveyed based on their race and mental illness. Hearing about the moral failing of an in-group member can cause discomfort if that individual identifies highly with their in-group members (29, 30). Therefore, for White individuals (most therapists in the United States and Canada), listening to stories about racism from BIPOC clients can lead to discomfort if those White persons identify highly with this group.

Such feelings of discomfort surrounding these topics may also be attributed to socialization in environments that do not have conversations about race. Thus, mental health professionals must resolve any anxieties they may have surrounding discussions about race, racism, and racial disparities because, unchecked biases can harm clients of color (31, 32).

### 4.3. Differential empathy for people of color influences diagnosis and treatment

Race has been shown to influence the level of empathy that individuals have toward each other. Several studies have demonstrated that those with similar skin color (Black or White) have greater empathetic resonance (33–35). Brain imaging furthermore reveals that White individuals perceive the pain of Black people as less painful than that of White people (33, 36). The evidence is compelling as it demonstrates that simply watching an individual being exposed to pain results in a measurable and quantifiable sensorimotor resonance, which is dependent on the racial similarity between the observer and the victim (37, 38).

These studies are important because reduced empathy on the part of White clinicians toward their Black patients may partially explain how mood symptoms in Black patients are often misinterpreted, or why psychotic disorders are overdiagnosed (39, 40). For example, instead of viewing a Black patient as “sad,” they are characterized as “mad.” Instead of being comforted because they are “afraid,” they are labeled as “paranoid.” These studies provide a mechanism for how what may traditionally be considered a reliable assessment of psychosis and schizophrenia across racial groups may in fact be biased.

### 4.4. History of medical racism and psychosis

These biases are not new but rather are rooted in our medical history. Racist discourse on mental health among medical professionals has occurred throughout the history of the United States. An early example of this can be found in the racist writings of a prominently published clinician, Dr. Samuel Cartwright, who taught at the University of Louisiana. In the article, *Diseases and Peculiarities of the Negro Race*, he identified/created two psychotic mental health conditions that he claimed to be unique to Black people. These conditions were “Drapetomania”—a disease that caused slaves to run away—and “Dyseathesia Aethiopica”—a disease that caused “rascality” in Black people both enslaved and free (41). He notes (p. 331–33), “They wander about at night, and keep in a half nodding sleep during the day. They slight their work, cut up corn, cane, cotton, or tobacco when hoeing it, as if for pure mischief. They raise disturbances with their overseers and fellow servants without cause or motive, and seem to be insensible to pain when subjected to punishment.” By pathologizing what were normal acts of survival and resistance by enslaved persons, he weaponized medicine in the service of upholding White supremacy.

Medical racism continues into the current time in its discriminatory treatment of Black people. For example, the practice of “race norming” for determining American football players' eligibility for settlement funds after traumatic brain injuries refers to the practice of generating different scales and thresholds for the assessment of cognitive functioning for White players than for Black ones. The norms used in the testing of decrement in cognitive function assumed that Black players have a lower cognitive function at baseline, and so must demonstrate a higher decline in cognitive function compared with non-Black players to qualify for financial settlements. This practice only ended in 2021 after public outcry (42). The NFL had defended this practice in the past, asserting its standards “relied on widely accepted and long-established cognitive tests and scoring methodologies.” As illustrated, race and racism contribute to the misdiagnosis of psychosis in African Americans with serious negative consequences for mental healthcare.

Notably, the care, treatment, diagnosis, and social understanding of psychosis in people racialized as Black in North America has a history of being fraught with issues that can confound the interpretation of patient data. This is because it is linked with the White supremacist roots in the very development of psychology as a scientific discipline and difficulty in identifying current racial myths, which may be anchored in the published literature as “scientific facts” (32, 43).

Before the 1960s, persons with schizophrenia were neither feared as criminals nor stigmatized as abnormal; in popular media, schizophrenia was more often associated with White women who developed psychosis due to the stresses associated with being a mother and homemaker (44), although this may not have been the conceptualization among those in the mental health community at the time. Even so, in 1968, a fundamental shift occurred subsequently transforming the meaning of psychosis in the minds of both healthcare professionals and the public. In that year, New York psychiatrists Walter Bromberg and Franck Simon coined the term “protest psychosis” to advance the idea that “Black power” sentiments drove “Negro men to insanity”, at which point schizophrenia became a condition used to pathologize Black people (44, 45). This weaponization of medicine was not a new phenomenon, rather only a continuation of the same war aimed at subjugating people by race. Not coincidentally did the emergence of codifying mental health as a tool to denigrate Black people occur just as the American Civil Rights movement was transforming society, inciting feelings of threat in many beneficiaries of the Jim Crow system (44). The purpose of redefining schizophrenia as a disorder to be feared was to support and preserve racial segregation and use the threat of mental illness to control agitators and punish social gains (44, 46). The race-based pathologization of psychosis that started in the 60s was exacerbated by legislative changes in the United States (de-institutionalization) between 1963 and 1965 that emptied psychiatric hospitals in the hope that discharged patients would be cared for in the community (47). But new medications were not simple cures for complex mental disorders and local communities were unprepared for the shift. Many former long-term patients whose families could not care for them were later found either on the street or in jails.

These events left their stain on the conceptualization of psychosis, which to this day continues to influence the understanding and perception of schizophrenia-spectrum disorders. Notably, from 1950 to 1996, the proportion of people who conceptualized those with schizophrenia-spectrum disorders as being violent increased by nearly 250% (48). The unexpected increase in perceptions of violence was confined to those who think of mental illness in terms of psychosis, and not for other conditions such as MDD, showing that this critical misperception about those with schizophrenia-spectrum disorders has become a socially learned bias.

The prism through which psychologists make diagnoses, which is initially based on the observation of behavior, is already tainted by socially learned biases (32). For example, a White psychologist interprets a remark about “people out to get me” as paranoid delusions while a Black psychologist may understand this as perfectly reasonable given that the patient has been stopped by police seven times in one month. Therefore, it is to be expected that racial disparities have been reported in the rates of diagnosis of these disorders (14). Upwards of 76% and an estimated 80% of licensed psychologists in the United States and Canada, respectively, are White (49, 50). Without lived experience to help understand the current social context, clinicians may have difficulty equitably diagnosing patients from cultures where they have little formal training. These unaddressed implicit biases around mental illness and psychosis with regard to other cultures also make it difficult to make comparisons across races.

It is through this lens that the recent increase in the diagnosis of schizophrenia-spectrum disorders in people of color should be considered (14). It has now been noted in several publications that providers interpret behavioral characteristics used in the diagnosis through their own racial or cultural biases, leading to misdiagnosis in Black individuals [e.g., (51, 52)]. Although studies from the United States show that the overall mental health of Black Americans was better or equivalent to that of Whites, with notably lower rates of alcohol and substance use than White groups, Black Americans in general have been diagnosed with higher rates of schizophrenia-spectrum disorders (12, 53, 54) for reasons that we now know may be difficult to disentangle from the initial weaponization of this diagnosis in the late sixties. The differences described are not genetic because, as previously stated, race is not a genetic construct (14).

A more nuanced reading of the literature, including new publications on genetics and socialization, points more to structural and identity-based exclusion as a primary cause that is affecting the variation in rates of schizophrenia-spectrum disorders among races (55). Therefore, it is with a critical eye, taking into account the history and politicization of schizophrenia-spectrum disorders and a lack of cultural competency of the primarily White clinician healthcare community who have been making these assessments, that one must view the literature.

### 4.5. Why Black people are overdiagnosed with schizophrenia-spectrum disorders

Olbert et al. (15) conducted a meta-analysis to determine the disparities in the diagnosis of schizophrenia between Black and White people in the United States using the DSM-III or later. Furthermore, they investigated whether the use of structured-interview assessments could reduce these disparities. They found that Black individuals were ~2.4 times more likely to receive a diagnosis of schizophrenia than White people and that the use of structured-interview assessments did not have a significant effect in reducing these racial diagnostic disparities (but a power analysis showed that there may be a small effect). The fourteen studies that were coded as having used structured interviews, conducted a clinical diagnosis using “structured- or semistructured-interview methods or validated symptom checklists” [(3), p. 106]. However, it is unknown if these interviews were culturally informed or whether they were conducted by culturally competent clinicians, and if they had been, whether this would have reduced racial diagnostic disparities. In this regard, as noted by Olbert et al. (15), simply addressing clinician bias and modifying structured-interview instruments will not be sufficient to overcome these racial diagnostic disparities. Other factors such as social, cultural, structural, and institutional disparities that impact Black people would also need to be addressed, as has been stated earlier in this paper.

Black people may be misdiagnosed with schizophrenia-spectrum disorders for several reasons. One proposed reason is a *healthy suspicion* (56), also known as responsive paranoia (57) or cultural mistrust (58). *Healthy suspiciousness* refers to “normative reactions” such as guardedness and mistrust that occur in response to discrimination (59). A study by Combs et al. (60) found that Black individuals endorse higher levels of subclinical paranoia when compared with White control groups. It was also found that perceived racism predicts cultural mistrust and non-clinical paranoia in Black people, and some paranoia may also be viewed as a healthy and adaptive strategy for Black people (59). Healthy suspiciousness may be misunderstood by mental health clinicians and considered to be delusional (61).

A second proposed reason relates to the consequences of stereotypes and implicit bias. Implicit bias refers to unconscious thoughts and feelings, which, due to lack of consciousness, are often not acknowledged or controlled by the person holding them. While overt acts of discrimination may have lessened in the United States, more subtle forms of discrimination, such as implicit bias on an individual and institutional level continue and may impact mental health clinicians' behaviors and choice of treatment (62). For example, these biases may lead to misperceiving Black individuals as “scary, violent, unreliable, less educated, and noncommunicative” [(63), p. 1123], thus interfering with a proper assessment. The study by Plaisime et al. (63) explored how the biases of White and Black healthcare providers in the United States may impact their diagnosis and treatment of Black male patients. Participants indicated that their perceptions were influenced by the largely negative portrayals of Black people in the media. White healthcare providers expressed fear and discomfort regarding working with Black male patients, and that they had very little contact with Black and other people of color often until graduate school. In addition, Black male patients were not offered certain treatments due to assumptions about low economic status and noncompliance. Black patients may also not be prescribed medications because of assumptions about drug use and addiction that are racially driven (64, 65). Furthermore, patients of color may have longer wait times for assessment and treatment (62, 64, 65), be approached condescendingly without consultation and collaboration, not receive a thorough assessment, and be denied interpreters if needed, as well as treatment options and access to family visits (62).

**Figure 2:**
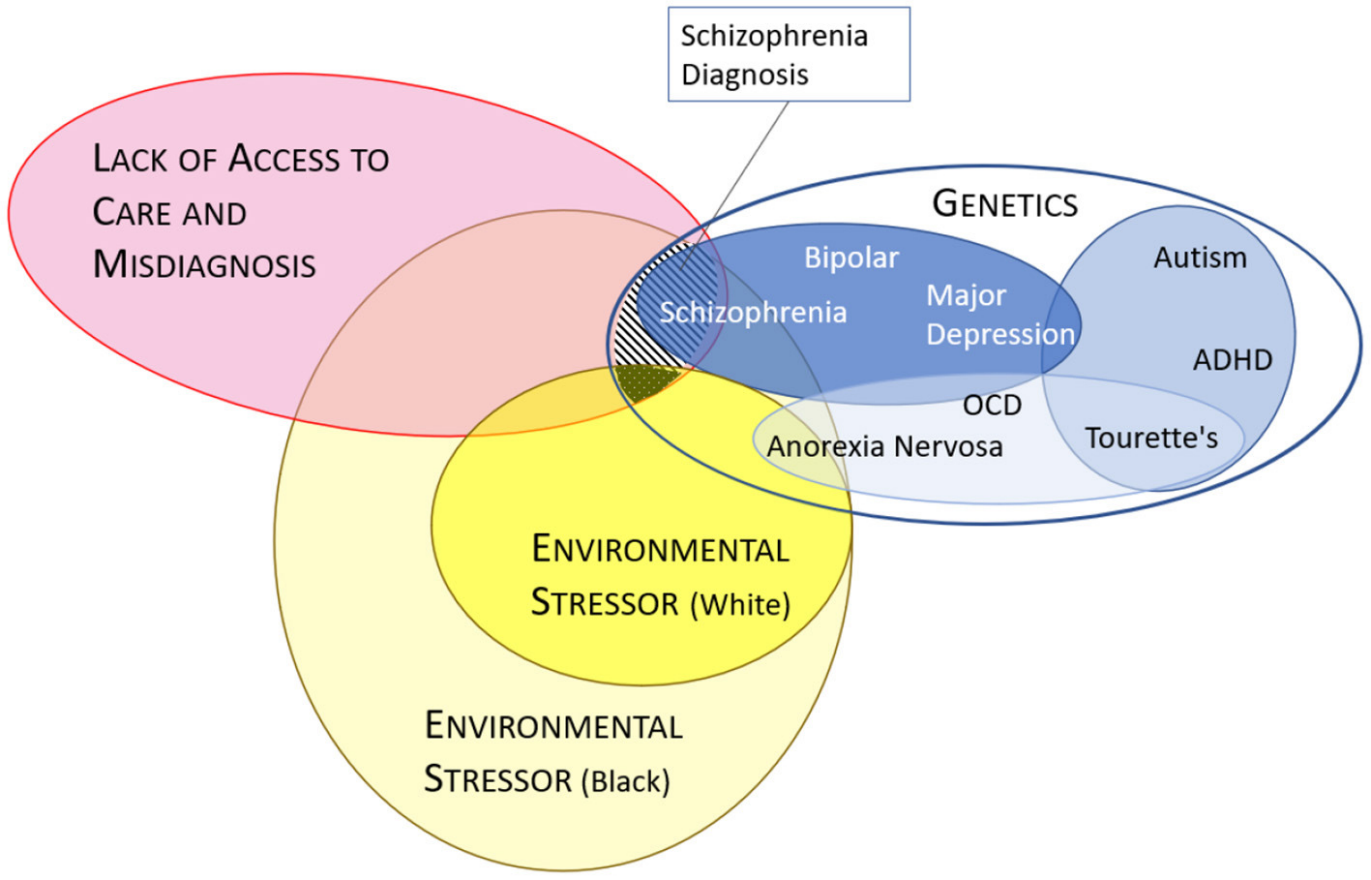
Potential factors leading to higher observed diagnoses of schizophrenia. Model of how factors leading to a diagnosis of schizophrenia can differ between Black and White individuals taking into account differences in environment, access to care, misdiagnoses and genetics (15). Stripped area represents diagnoses of schizophrenia in Black populations which has been observed to be greater than in White populations (dotted area). The blue ovals (genetics) are based on a risk model from the Cross-Disorder Group of the Psychiatric Genomics Consortium (3). Misdiagnosis and lack of care (red oval) appears to affect racialized individuals (i.e., Black) more often than White individuals (intersecting orange areas).

Research by Strakowski et al. (66, 67) has shown there to be a clinician overemphasis on the relevance of psychotic symptoms and an underemphasis on mood-related symptoms in the diagnosis of schizophrenia-spectrum disorders in African Americans. This pattern was also found in a more recent study conducted by Gara et al. (39), which showed that, when compared with Non-Latino Whites, African Americans who screened positive for major depression (moderately severe to severe depression) were significantly more likely to receive a misdiagnosis of schizophrenia. Gara et al. (68) found no significant difference between blind ratings for the severity of depressive and manic symptoms between African American and White individuals; however, African American patients were assigned higher ratings of psychosis. This suggests that in Black patients, clinicians tend to overemphasize psychotic symptoms, and diagnoses may be “skewed” toward schizophrenia-spectrum disorders, even though they exhibit similar levels of depressive and manic symptoms as White individuals (68). Misdiagnosing patients through the influence of bias and stereotypes can harm patients psychologically or socially (14), as per Figure 2.

Finally, the failure to consider the role of spirituality and religious beliefs may lead to an inaccurate diagnosis of schizophrenia-spectrum disorders [e.g., (50)]. Results from the study by Peltier et al. (69) showed that for African Americans, the probability of a schizophrenia-spectrum disorder diagnosis decreased when unusual experiences were viewed by the clinician as spiritual or paranormal. A religious person who says that they have “heard the voice of God” should not automatically be assumed to have had a psychotic experience. Clinicians cannot assume that all visual and auditory hallucinations indicate the presence of a schizophrenia-spectrum disorder (69), as a culturally informed assessment must include the consideration of diverse spiritual beliefs and practices (69, 70), particularly in relation to hallucinations and religious-themed delusions.

It is important to note that schizophrenia is a diagnosis of exclusion, meaning that clinicians are expected to rule out other causes for symptoms and other diagnoses before assigning a diagnosis of schizophrenia (39, 68). This does not appear to sufficiently occur for Black communities due to assessment bias in the diagnostic process, as well as failure to account for the social and environmental risk factors and cultural factors that contribute to the development of symptoms (16). According to Gara et al. (68), understanding the impact of racial bias in clinical assessment is crucial and can reduce racial disparities in healthcare.

### 4.6. Genetics, race, and schizophrenia

The conflation of race, ethnicity, and genes has led to confusion around the understanding of findings that have been interpreted to mean that certain races are biologically inclined to have an increased incidence of schizophrenia. New data however shed light on the relationship of schizophrenia with specific genes (3, 4, 71). These data indicate that schizophrenia exists in a genetic category that places it in the same group as bipolar disorder (Figure 2). This categorization underscores that the diagnosis of schizophrenia, like most mental health disorders, does not rely on objective findings such as blood tests or brain scans and is based on observations from patients, family members, and the diagnosing physician. Lack of appreciation of the linkages among mood and psychotic disorders has resulted in schizophrenia being treated as if it were (i.e., by DSM-5) more disconnected from other psychological disorders than is warranted by the genetic data, which affects treatments and clinical care as well as the cultural and social meaning of having a diagnosis of schizophrenia or psychosis (72).

Two recent publications have been able to locate ultra-rare variations in several genes that increase a person's risk of developing schizophrenia. The basis for one of these studies was a whole-exome sequencing of about 24,000 persons from diverse global populations diagnosed with schizophrenia and 97,000 without schizophrenia (4). The nature of the discovered genes confirms that schizophrenia is a disorder of neuronal communication. The genomic regions that were identified are in ion channels, transporters, and genes regulating expression, primarily only active in neurons of the brain. These genes have their effects on mechanisms such as synaptic structure and some of the identified variants code for genetic mutations that result in a truncated version of the affected protein. If any given individual has a 1% chance of being diagnosed with schizophrenia over a lifetime, having one of the identified mutations can increase the risk by four to 50 times depending on the genetic mutation (4, 73).

Although fewer than one in 10,000 individuals carry any of these ultra-rare higher-risk mutations, simply having a variation in one of the ten most identified genes could boost the risk of contracting schizophrenia, and having combinations of these variants may further stack the odds toward the development of schizophrenia (4, 73).

Genes in the glutamate neurotransmitter pathway including GRIN2A, SP4, and GRIA3 are among the high-risk genes, which confirms the suspicions of researchers. It has long been observed that pharmaceuticals that antagonize receptors in the glutamate pathway including ketamine and PCP are capable in certain doses of triggering symptoms that look like schizophrenia (4, 73).

These breakthroughs in understanding the genetic risk factors for schizophrenia help clarify origins and point away from a risk model based on simplistic racial differences in diagnoses. None of the identified genes are thought to have a direct association with skin shade, a key basis for racial classification. There is no reason to assume a biological or genetic cause for the observed higher prevalence of schizophrenia-spectrum disorders in specific minoritized groups (74), rather strong evidence exists from other studies against a race-based genetic explanation of risk.

These studies examined the same ethnic groups that had vastly different incidences of schizophrenia-spectrum disorders depending on their geographic location. In one study of ethnically Norwegian migrants in the United States state of Minnesota, higher rates of schizophrenia were found in the migrant than in the original population in Norway. Similarly, ethnically similar groups originating from Trinidad, Suriname, and Jamaica had much higher rates of diagnosed psychotic disorders in Western Europe than in their home countries (74). Further empirical research shows, also, that adjusting for environmental risk factors such as social disadvantage and linguistic distance—markers associated with increased odds of psychotic disorders—led to equal risks between ethnic minority groups and White majorities in several EU countries (55). Taken together, these studies do not support the theory that some races or ethnic groups have an increased gene-based propensity to develop schizophrenia-spectrum disorders (55, 74).

Although there are now genes associated with the risk of developing schizophrenia, these do not directly correspond to higher risk by race, as race is a sociopolitical and not a genetic classification. Notably, since belonging to a certain race corresponds to an environmental risk of poor mental health (i.e., social disadvantage), or a tendency to be medically misdiagnosed, an association between race and schizophrenia-spectrum disorders may be expected to be observed (Figure 2).

### 4.7. Black people may have increased vulnerability due to more PTSD

In addition to overdiagnosis, Black people may have an increased vulnerability to schizophrenia-spectrum disorders due to the higher prevalence of posttraumatic stress disorder (PTSD) and trauma (75, 76), which is a well-established risk factor in the etiology of psychosis (77). The story of Josh Marks (Table 3) is illustrative of many of the issues facing Black people who struggle with severe mental illness.

**Table 3:** Case study: The tragic suicide of a master chef rising star.

**Description**	**Common issues arising in care pathways for psychosis and schizophrenia**
Josh Marks was a rising star, having placed as the first runner up in the renowned and beloved cooking competition “Master Chef”, when in September of 2012 he began to behave strangely. By January 2013, he began telling his family he was battling evil spirits, and shortly thereafter was hospitalized from a car accident. While in the hospital after behaving erratically; he was strapped to a bed and put on a 72 hours hold. That evening the doctors suspected his increasingly bizarre behavior was due to psychosis and his family was able to arrange a short stay at Rush Medical Hospital.	*The patient's family lacked mental health literacy about psychosis*.
Psychiatrists told Marks' mother that her son was bipolar and was experiencing psychotic episodes. He was sent home with only a prescription for lithium and instructions to watch him closely. His mother was confused as he had never shown any sign of poor mental health. She was unable to find help as every place she called told her that without insurance they would not be able to help. She was eventually able to enroll him in a Cook County Medicaid program (Affordable Care Act). However, even after approval, there were problems because the program was so new, that many providers had not started accepting this type of insurance.	*No beds, and warehousing instead of therapy. Funding for mental healthcare services have been cut everywhere; Illinois's per capita spending ranked 36th nationally in 2010*.
Marks' trajectory took a downward spiral. One night he asked his mother if she too heard all of the voices he was hearing in his head. Later in the car together, he leaped out of the vehicle and just started screaming. Terrified, his mother could only watch as he sped down the street. At a loss, she called 911. By the time officers arrived, he was again calm, but unable to communicate and laid down on the sidewalk. Again, he was taken to the hospital, this time to St. Bernhards, and again they examined, prescribed additional medication, and discharged him.	*Law enforcement used to address the acute mental health crisis. Law enforcement uninformed as to how to address acute mental health crises*.
By July 2013, after wandering aimlessly for hours, in a misguided attempt to quiet the voices in his head while sitting in his car, Marks shot himself in the ear. He survived and was found by Chicago University campus police after he placed a 4 am emergency call for help. They found him with cuts from bullet fragments all over his face.	*Psychotic break misinterpreted by police because of race*.
The police allege that Marks attacked them when they asked him what was wrong and that he tried to take their gun. During the arrest, he was beaten with a baton, tackled to the ground, and sprayed with pepper spray; he babbled incoherently throughout the incident. The police violence left him with a hematoma and a broken jaw, which required surgery. He was charged with felony aggravated assault against a police officer and his bail was set at $150,000. Still undertreated for psychosis, he was remanded to the Cook County jail. His jaw was still wired shut.	*The final diagnosis came late and without resources to treat the disorder*.
A few weeks after his release from jail, his mother discovered that Mercy Hospital and Medical Center accepted CountyCare insurance. At no cost to the family, the hospital provided a two-week inpatient stay and agreed to eight weeks of outpatient therapy: Upon his release from the hospital, however, Marks was given discharge papers which included a final additional diagnosis: paranoid schizophrenia. This diagnosis was devastating for the whole family, who took on the responsibility to watch Marks around the clock. But on October 11, 2013, his mother found him lying on his back, staring up at the heavens with a gun beside him. There was only a small wound, a bullet hole in his head. He was gone [Adapted from Smith (78)].	*The family required to take on the role of an inpatient facility. The lack of treatment was fatal*.

A meta-analysis by Bailey et al. (79) showed that trauma experienced in childhood may increase the likelihood of hallucinations and delusions. In addition, social marginalization is a known risk factor for developing schizophrenia and psychosis (80), as are environmental factors such as neighborhood conditions—the neighborhood index of multiple deprivation (IMD) significantly predicted psychosis and depression, especially psychotic symptoms with paranoia (81). Black individuals are at high risk for traumatization, including racial trauma, due to discrimination and racism at individual, societal, and structural levels (26). An umbrella review by Varchmin et al. (82) found that one of the most significant social risk factors for psychosis was a vulnerability to racist discrimination, particularly for migrants in low ethnic density areas, minoritized individuals, and Black individuals.

The cumulative effects of racism can exacerbate any genetic predispositions and contribute to PTSD and racial trauma that can go untreated due to institutional racism and lack of access to appropriate care (26, 83). Structural racism, experienced “disproportionately” by Black people, confers social disadvantage through neighborhood factors, discrimination, and collective stress and trauma, which may place them at risk for developing schizophrenia-spectrum disorders (16, 80). Adequate mental healthcare needs to also address these social and environmental factors that include everyday racism, structural racism, and their impact on informing symptoms.

Misdiagnosing patients with schizophrenia-spectrum disorders has many serious implications. If misdiagnosed, patients do not get the correct treatment for their symptoms and conditions. For example, patients with bipolar disorders who are misdiagnosed as having schizophrenia may not be prescribed mood stabilizers (e.g., lithium) to address symptoms of mania. Depressed patients who receive antipsychotic medications due to misdiagnosis must endure serious side effects such as weight gain, metabolic syndrome, diabetes, and movement disorders, which can cause unnecessary physical harm and health consequences (39). Misdiagnosis also contributes to potential lifelong stigma and hopelessness (8), lower expectations for prognosis, and restricted options for treatment (39, 68).

## 5. Black experiences in medical contexts

### 5.1. Access to care in the first episode

Overall, people of color experience discrimination in access to and the quality of healthcare at both individual and structural levels (26). The United States cohort study of 3,017 privately insured patients (age 10–21 years) found that White patients were more likely than Black and Hispanic patients to be given behavioral health diagnoses and treatment before the identification of first-episode psychosis. Controlling for socioeconomic variables did not change these observed racial/ethnic disparities. Specific to first-episode psychosis, newly diagnosed youth of color do not see a mental healthcare professional unless they are admitted to the emergency room (84).

There is evidence that people of color are receiving less mental healthcare in the year leading up to their diagnosis of first-episode psychosis (84). Usually, there are subtle attenuated prodromal symptoms (85) that occur before a first-episode psychosis, which are critical for detecting individuals at clinically high-risk for psychosis (84, 86). Timely or early intervention at this stage can reduce the duration of untreated psychopathology, prevent the worsening of symptoms, and improve clinical prognosis (84, 87, 88). Historically, efforts to recruit clinical high-risk individuals for research studies have resulted in the majority of White samples (89), while first-episode psychosis research studies report an overrepresentation of Black patients. These data suggest that Black patients are more likely to access care once the overt symptoms of psychosis have emerged, and efforts are needed to improve their access to clinical high-risk programs. According to Heun-Johnson et al. (84), unequal access to healthcare in the prodromal period may worsen long-term treatment outcomes after diagnosis. These studies demonstrate that it is essential to address racial/ethnic disparities in accessing healthcare so that people of color can receive early intervention, which is critical for a good prognosis (16).

### 5.2. Overmedicated due to exaggerated fears of dangerousness

When in treatment, Black patients are more likely to be overmedicated due to being stereotyped as “dangerous” by clinicians (16, 90). Segal et al. (90) found that patients' medication status was impacted by their race. Specifically, African American patients were more likely to be prescribed psychiatric medications and administered more high-potency antipsychotic medications at higher doses. They were also given significantly less time for evaluations by clinicians. Furthermore, Black Americans are also often prescribed older medications or first-generation antipsychotic medications, which can have severe side effects such as tardive dyskinesia and extrapyramidal side effects (91, 92). Herbeck et al. (91) found that in comparison to White Americans, African Americans were significantly less likely to be given second-generation antipsychotics, which are generally considered to be a better treatment for schizophrenia-spectrum disorders. In particular, young, male African Americans, or those with schizophrenia, were less likely to be given second-generation antipsychotics. This is of concern because younger patients may benefit from second-generation antipsychotics because of their decreased side effects (91). In addition, Herbeck et al. (91) found that individuals without health insurance were less likely to be given second-generation antipsychotic medications, noting that 10% of African Americans did not have health insurance in comparison to the 3% of White Americans. Similarly, Cook et al. (92) found that Black individuals in high-poverty communities were more than two times as likely to be treated using high-potency first-generation antipsychotics. These racial disparities in treatment continue even though second-generation antipsychotics have been prescribed for over 30 years (92).

Overmedication and the side effects associated with first-generation antipsychotics can negatively impact health and may contribute to decreased compliance with medication (91). This may then result in coercive techniques by healthcare providers to gain compliance. For example, Knight et al. (93) found that Black Canadians of Caribbean or African descent were more likely to receive coercive referral and treatment (through police, ambulance, and court) compared with White Canadians, and were at greater risk for legal coercion and detention for treatment. These disproportionate rates of coercive referrals of Black individuals make a strong case for researching how “authority figures” determine dangerousness in the case of Black patients, especially those with first-episode psychosis (93).

Such discriminatory practices are indicative of racial bias and institutional racism and are highly problematic because coercive treatments often foster cultural mistrust and create a poor patient–clinician relationship. Coercive approaches can also be very harmful, as techniques such as involuntary hospitalization and mandated medication may induce trauma in the patient and their families, leading to greater distrust and avoidance of mental healthcare in the future (94, 95). It is abundantly clear that how Black patients are assessed for treatments needs to be changed. Along these lines, Segal et al. (90) showed that when clinicians in emergency services make efforts to engage patients in treatment, overmedication decreases, underscoring the critical need for engaging Black patients in the evaluation and treatment process.

### 5.3. Pathways to care vary by ethnoracial group

“Pathways to care” refers to the sequence of interactions, both positive and negative, with individuals, organizations, or events, when an individual or their family attempts to seek help (96, 97). Ideally, each person will have a clear pathway for receiving appropriate care; however, this concept is also used to assess delays in help-seeking and treatment to better understand ways of providing early intervention (98). People of different races and socioeconomic statuses have different pathways that provide them with access to care (96, 99). “Aversive pathways” to specialized care for first-episode psychosis are associated with delays in treatment resulting in longer periods of untreated psychosis, poor engagement with treatment, and poorer outcomes. However, there is little research conducted on the “unique pathways” to care for Black individuals seeking care for schizophrenia-spectrum disorders (16, 96).

A qualitative study of coordinated specialty care for schizophrenia-spectrum disorders among Black Americans with first-episode psychosis found that before the onset of psychosis, participants reported exposure to childhood trauma, diminished social functioning, and greater contact with law enforcement that families described as “overly aggressive” (96). In addition, many prodromal experiences persisted following the onset of psychosis and were associated with delays in treatment and difficulties with navigating services, leading to increased delays in receiving coordinated specialized care. These barriers, which were specifically associated with longer durations of untreated psychosis for African Americans, had been previously described in a qualitative study by Bergner et al. (100). First, they found that healthcare professionals tended to misattribute symptoms of psychosis as depression, stress, or drug use. Second, they initiated treatment based on the presence of positive symptoms of psychosis, waiting for an increase in the severity of symptoms. Third, they required symptoms to reach a certain threshold, using issues of patient autonomy as an excuse. And finally, they allowed systemic factors such as lack of social support, lack of affordable services, and difficulties with scheduling appointments to interfere with the delivery of adequate care (100). In other words, healthcare professionals were providing racially biased and substandard care targeted specifically at Black individuals.

For Black communities, there is an extreme and damning gap between those who require mental health services and those who actually receive timely expertise and compassionate services. According to Tambling et al. (101), a full 70% of those who require treatment fail to access appropriate services (101, 102). The picture in Canada is similarly bleak, with a national survey finding that 42.2% of those seeking care for schizophrenia had difficulty accessing services “often” or “just about every time” (103).

## 6. Role of law enforcement

### 6.1. Disturbing statistics

Disparities in pathways to care may arise when law enforcement is involved. Young Black people with psychotic symptoms may be stereotyped as dangerous, and police may be called to deal with patients, putting them at increased risk of harm. However, people experiencing a mental health crisis are more likely to be victimized than cause harm to others (104).

Individuals with untreated severe mental illness are involved in at least one in four and as many as half of all fatal police shootings (105). Saleh et al. (106) included demographics and the presence of mental illness in their investigation of independent databases of killings of civilians by police in the United States. They found that in 2015, 23% of people killed by police showed symptoms of a mental illness. Such tragedies have resulted in a call for action from Black communities and from organizations such as The Centre for Addiction and Mental Health (107), which holds the position that racism and anti-Black racism exacerbate the interactions of individuals experiencing mental illness with law enforcement that compounds their mental health crisis.

Implicit bias and racism on the part of the police are a cause for violence against Black individuals in need of mental healthcare (108, 109). In the United States, the twenty first century Cures Act, passed by Congress and signed by President Obama in December 2016, included a mandate for the United States Attorney General to collect and report data on the role of serious mental illness in fatal law enforcement encounters. The Bureau of Justice Statistics overhauled its system for collecting law enforcement homicide data and at that point resumed reporting arrest-related death statistics. Using the new methodology approximately doubled the number of arrest-related deaths that were verified and reported by the Department of Justice. However, the role of mental illness in them has not yet been reported (105).

### 6.2. Disturbing examples

There are many problems with including police in situations involving a mental health crisis, as illustrated in Table 4. In all of these cases, police used disproportionate force resulting in tragic deaths, which is already a problem facing Black people (110). These shootings are driven by racism and fear, which has now become a part of the public discourse (20, 111, 112). Simply being Black and being associated with a mentally ill person is perceived as a danger, as evidenced by the case of an unarmed Black therapist shot while laying down with his hands in the air trying to prevent his autistic White patient with a toy firetruck from being shot by police (113). In addition, in many cases, Black people coping with schizophrenia-spectrum disorders are not killed or taken to a hospital but are simply criminally charged and jailed, as we saw in the case of Grace Terry, where today the United States prison system is the largest provider of mental health services in the world (114). Black Americans are incarcerated in state prisons at nearly five times the rate of White Americans and makeup 38% of the total prison population (115, 116), while one study in Ontario also found disproportionately higher incarceration of Black individuals [Black men are five times more likely to be imprisoned than White men; (117)]. Notably, one study found that in a sample of 109 urban, low-income, predominantly African American patients hospitalized for first-episode psychosis, an outsized 57.8% had been previously incarcerated, and for predominantly non-violent crimes (17).

**Table 4:** Fatal examples of police interactions with racialized psychosis patients.

**Name**	**Incident**	**Response**
Clive Mensah	A 30-year-old unarmed Black man from Mississauga, Canada, died after being tasered by police even though he followed police orders. He had mental health issues, “possibly schizophrenia” and was “frequently observed speaking loudly to himself.” On the night of his death, calls were made to police that he was making a lot of noise and walking on the street “...swinging his arms, and screaming and yelling” (118).	Although police acknowledged that he needed help, they did not call a mental health crisis team. Instead, police tasered and pepper sprayed him, and he died.
Ejaz Choudry	A 62-year-old Muslim man with schizophrenia from Mississauga, Canada, whose family called a non-emergency line looking for help because he had stopped taking his medication. He appeared confused and had a knife. During this three-hour interaction, he did not receive the required care and was shot dead by the tactical unit (119, 120).	Instead of a crisis team, the police arrived, and despite being told that he did not understand much English, told him to drop his knife.
Regis Korchinski-Paquet	The family of a Black Indigenous woman from Toronto, Canada, made an emergency call looking for help due to a domestic disturbance involving her and her brother (121).	Police could not handle this crisis, and she fell from her balcony to her death.
D'Andre Campbell	A young Black Canadian man with schizophrenia called the police requesting to be taken to the hospital. Instead of the mental health crisis intervention team, police officers were dispatched (122).	Although known to police as someone who had reached out in crisis before, police used stun guns and then fatally shot him.
Ricardo Muñoz	The sister of a 27-year-old man with a diagnosis of paranoid schizophrenia from Pennsylvania called the police for help because he seemed very agitated and had not taken his medication (123).	Police fatally shot him multiple times.

### 6.3 Weaponization of medicines by law enforcement

Substances that are intended to be therapeutic can be used to control or harm people of color, and this is also referred to as “weaponized medicine” (124). The first account in the literature of this was in 1928, in the *Journal of the American Medical Association*, which reported that in Hawaii, an ethnically Japanese handyman and chauffeur had been arrested as the chief suspect in the kidnapping and murder of a young White boy. A police surgeon injected him with a substantial dose of hyoscyamine to extract a confession, which he later recanted, and another person was later found to be the perpetrator. This approach was later used on hundreds of convicts.

Recently, two tragic incidents in 2020 involving law enforcement raised concerns over the use of anesthetic ketamine—a powerful sedative normally used in hospitals—as a law enforcement tool to subdue suspects in the field (124, 125). Over a four-day period in Aurora, Colorado, 23-year-old Elijah McClain and 25-year-old Elijah McKnight were both given doses of ketamine in separate police incidents. McClain went into cardiac arrest and died several days later. McKnight was hospitalized on life support but survived. Video footage of both incidents shows that neither McClain nor McKnight was resisting arrest when the ketamine was administered.

These incidents demonstrate that the lawlessness of law enforcement in punitive interactions with Black persons is ongoing. Not only are Black people targeted simply because of their race, but psychosis-inducing substances are also being used to create the veneer of an excuse, which may be justified through myths around the danger of Black psychosis (44). In these cases, the police used the label “excited delirium” to justify the subjugation of suspects with ketamine. However, the diagnosis of excited delirium is not recognized by major medical organizations such as the American Medical Association (126). Furthermore, police officers are not trained to diagnose any sort of delirium, rather this role belongs to medical personnel (127). It may be more accurate to describe the incident involving McClain as an extra-judicial murder using psychosis-inducing drugs, as the circumstances were suspicious. It is now known that McClain only weighed 143 pounds at the time of his arrest yet was given a ketamine dose while handcuffed, he was given a dose for a 200-pound man (128). In 2022, two years after his death, two paramedics and three police officers were indicted by a Colorado grand jury on charges of manslaughter and criminally negligent homicide (129). Despite these issues, the diagnosis of “excited delirium” continues to be used throughout the United States to justify the use of ketamine and to explain deaths in police custody, especially the deaths of young Black men (130).

## 7. Addressing the issues

Given the unique issues faced by Black people with early symptoms of schizophrenia-spectrum disorders, solutions must be tailored to address their needs, which are complicated by systemic racism in healthcare and law enforcement. Critical issues to address include (1) improving diagnostic accuracy which includes educating psychologists about the unique cultural history and stigmas around psychosis as well as its genetic origins, (2) public education in communities of color to increase mental health literacy, (3) improving interventions surrounding law enforcement for mental health crises, and (4) anti-racism training for mental health clinicians. Examples of how to accomplish this are shown in Table 5.

**Table 5:** Examples of how to reduce racial disparities in mental healthcare pathways for Black individuals.

**Improving diagnostic accuracy**	
*Structured interviews*	• Structured interviews have proven value in improving diagnostic accuracy and can be used in real clinical settings (131).
*Racial/ethnic matching*	• Methods used in the National Survey of American Life investigating the nature, severity, and impairment of mental disorders in Black and White populations used racial and ethnic matching of interviewers and respondents (132).
**Targeted public education in communities of color**	
*Mental health literacy*	• Norway's Treatment and Intervention in Psychosis program, Australia's *beyondblue* initiative for depression and related disorders, as well as Nuremberg's community campaign for depression, represent examples of successful mental health literacy programs (133).
*Public awareness campaigns*	• One comprehensive mental health awareness program (the Depression is Real Campaign) launched in Louisville, KY (134) developed culturally informed communication about the nature of depression, its symptoms, and treatment for Black communities. A similar approach could be used to raise awareness about psychosis.
*Word-of- mouth*	• *MindStylz* is a collaborative project between hair stylists, barbers, and the Ethnic Diversity Task Force of the Connecticut Psychological Association (135). It's mission is to promote mental health awareness among hair stylists, barbers, and their clients in communities of color. *MindStylz* helps by providing education and resources to hair stylists and barbers for their clients.
*Church-based approaches*	• In 2022, the first randomized trial of church-based counselling centres for depression counseling in African Americans started. The idea is to improve care in economically disadvantaged areas (136).
**Revisiting the role of law enforcement**	
*Training law enforcement*	• Police in Louisville Kentucky worked with National Alliance on Mental Illness (NAMI) to reduce the harm caused by mental health crisis calls. Outcomes of a special police crisis intervention team (CIT) were reviewed over a year and compared with available pre-CIT statistics. The arrest rate for the CIT was lower than non-CIT runs while the occupancy of the local mental health unit in the jail stayed about the same (~1,100 patients/year), and referrals to intense psychiatric services (e.g., Central State Hospital) significantly dropped (from 53% in 2001 to 26.8% in 2004).
*Alternatives to police involvement*	• In both the United States and Canada, traditional law enforcement responders are being replaced with healthcare workers for some emergency calls. Previously, Denver 911 operators only directed calls to police or fire department first responders, but the Support Team Assistance Response (STAR) pilot program created a new track for directing emergency calls to a two-person medical team as did the Vivec Research Team in Ottawa (137, 138).
**Anti-racism and anti-bias training for mental health clinicians**	
*Anti-racism training*	• In the MGH/McLean Psychiatry residency program, the Division of Public and Community Psychiatry developed a curriculum addressing racial inequities in mental health, particularly those experienced by African Americans. Training discussing racism in formal didactics integrated into the required didactic curriculum was positively rated by participants (139).

### 7.1. Improving diagnostic accuracy

A more deliberate diagnostic process through greater use of the structured clinical interview for schizophrenia-spectrum disorders could reduce reliance on stereotypes and help minimize misdiagnoses [e.g., (3)]. Furthermore, when possible, the benefits of racial and ethnic matching should be considered, as many clinicians do not have the proper training to work effectively across race, ethnicity, and culture (32). For example, a Black person may be cautious or seem reluctant to share details with clinicians from other racial groups due to realistic mistrust that can then be misinterpreted as pathological paranoia.

### 7.2. Public education

Increasing mental health literacy at the community and population level can improve the early detection of and intervention for psychological disorders (140). Public awareness educational campaigns aimed at the general public can be beneficial in increasing awareness of the nature of schizophrenia and its treatment, although these campaigns should be a continuing process, as single campaigns usually do not have much effect (141). Specific messages disconnecting schizophrenia from violent behavior should be crafted as there appears to be a widely held misconception that they are linked (48). Key components of public education can include an emphasis on increasing positive word-of-mouth from trusted leaders in communities of color where long-term relationships are critical. Church-based interventions are effective strategies for reaching Black communities as these resources are connected to a trusted source (142).

### 7.3. Improving interventions surrounding law enforcement

As previously noted, people with severe mental disorders are much more likely to be killed by police, and the risks multiply when the victim is Black. Therefore, there is a need to address this problem at the level of law enforcement (108, 143, 144). Training for law enforcement on how to recognize and manage mental health crises is essential, but there is also a need for the formal separation of mental health interventions from policing. Police are not mental health providers, and armed persons in uniform should not be the first point of contact for people experiencing psychotic symptoms, especially Black people who are often already highly fearful of police (145, 146). Alternate first responders should be enlisted for crisis intervention and wellness checks as per examples in Table 5 (138, 147).

### 7.4. Anti-racism training

Organizations serving racialized groups can frame their actions and practices in anti-racist and anti-oppressive philosophies to be more responsive to the populations they serve (20). This can result in better care for racialized groups while addressing the issue of racism and oppression in mental health and medical services (148–150).

## 8. Conclusion

Black people in the early phases of a schizophrenia-spectrum disorder face barriers to proper diagnosis, impediments to proper care, greater mistrust of mental healthcare systems, and violence from law enforcement. Psychologists and psychiatrists are often the gatekeepers to proper care for these individuals. The clinical treatment of mental disorders is dependent on cultural norms, and the empathetic, culturally informed diagnosis of mental health professionals. Timely awareness of the significance of culture can make a difference in the pathways to care for Black individuals suffering from psychosis and correct any mistaken interpretations of psychopathology (2, 14). Failure to act in accordance with this can result in misdiagnosis and poor outcomes for those served.

While the research, training, and application of culturally responsive assessment and diagnosis have increased over the last several decades, there is a continued need for a focus on training culturally responsive clinicians. The skills of a culturally responsive clinician are similar to those of an effective clinician more broadly, developing cultural humility (to avoid mistaking difference for inferiority), compassion (for self and others), and critical thinking skills (the process of continually questioning one's assumptions and biases). Clinicians who attend to these principles achieve the overarching goal of every clinician: client wellbeing (32, 151). With this in mind, multifaceted approaches are needed to holistically address these problems that include interventions in the community as well.

## Author contributions

SF and MW: conceptualization—ideas formulation or evolution of overarching research goals and aims, methodology, development or design of methodology, creation of models, project administration—management, coordination responsibility for the research activity planning, and execution, supervision—oversight, leadership responsibility for the research activity planning and execution, and including mentorship external to the core team. SF, AKR, TM, and MW: investigation—conducting a research and investigation process, specifically performing the experiments, or data/evidence collection, writing—original draft, preparation, creation and/or presentation of the published work, specifically writing the initial draft (including substantive translation), and writing—review and editing. SF: visualization—preparation, creation and/or presentation of the published work, and specifically visualization/data presentation. All authors contributed to the article and approved the submitted version.
